# Cryptosporidiosis should be designated as a tropical disease by the US Food and Drug Administration

**DOI:** 10.1371/journal.pntd.0008252

**Published:** 2020-07-02

**Authors:** Robert K. M. Choy, Christopher D. Huston

**Affiliations:** 1 PATH, San Francisco, California, United States of America; 2 University of Vermont Larner College of Medicine, Burlington, Vermont, United States of America; Center for Biologics Evaluation and Research, Food and Drug Administration, UNITED STATES

Although the protozoan parasite *Cryptosporidium* spp. was recognized as a human pathogen in 1976 [1], it was only after a major outbreak in 1993 that sickened more than 400,000 and killed 69 in Milwaukee, Wisconsin, United States of America [2], that its impact as a diarrheal disease-causing pathogen began to be widely appreciated. *Cryptosporidium* has since been recognized as the most frequently identified pathogen in US waterborne outbreaks [3]. However, because otherwise healthy people recover spontaneously, it continues to be viewed in the US primarily as an opportunistic pathogen causing chronic diarrhea in AIDS patients and other immunocompromised people. In 2013, publication of the first results from the Global Enteric Multicenter Study (GEMS) highlighted the impact of this parasite among children in low-resource settings (LRS), including seven African and Asian countries. *Cryptosporidium* was found to be one of the three most important diarrhea-causing pathogens in children under 12 months old and the most common cause of mortality due to moderate-to-severe diarrhea among 12- to 23-month olds [4,5]. Subsequent epidemiological studies reinforced these findings, including MAL-ED (Etiology, Risk Factors and Interactions of Enteric Infections and Malnutrition and the Consequences for Child Health and Development) and more recent surveys by the Global Burden of Disease (GBD) and the GEMS-1A follow-on study to GEMS. At eight Asian and African sites, the MAL-ED study found *Cryptosporidium* was among the five most important pathogens causing diarrhea in community clinics [6]. The GBD study estimated that globally *Cryptosporidium* is responsible for more than 44.8 million episodes of diarrhea and 48,000 deaths annually [7]. GEMS-1A confirmed and extended the findings from GEMS by showing that *Cryptosporidium* was associated with a greater than 2-fold increased risk of death among 12- to 23-month olds with moderate-to-severe diarrhea [8].

Despite this substantial and well-documented burden, the impact of cryptosporidiosis is still under-recognized by the wider global health community: Cryptosporidiosis is not designated as a neglected tropical disease (NTD) by the World Health Organization nor is it included on the list of tropical diseases eligible for a priority review voucher (PRV) from the US Food and Drug Administration (FDA). We highlight below the reasons why cryptosporidiosis strongly deserves inclusion on the FDA list of tropical diseases and how this could help stimulate development of improved therapies.

The current toolbox of interventions against cryptosporidiosis is severely limited. There are no approved vaccines, and there is only a single anticryptosporidial drug, nitazoxanide. Although nitazoxanide reduces the duration of illness in immunocompetent adults, it is inadequate for the populations with the greatest need. Nitazoxanide is no better than placebo in severely immunocompromised subjects, such as HIV patients [9], and only modestly effective in malnourished children [10]. The development pathway for anticryptosporidial drugs is especially challenging, given the lack of a simple in vitro culture system and the need to demonstrate safety and efficacy in young children. Nonetheless, based on recent successes in high-throughput phenotypic screening to identify *Cryptosporidium* growth inhibitors [11] and in developing antimalarials targeted for use by pregnant women and children [12], similar approaches have an excellent chance of identifying safe and effective treatments for *Cryptosporidium*. Indeed, as summarized in Table 1, multiple high-quality leads have recently been identified, making adequate pharmaceutical company interest one of the most important remaining impediments.

**Table 1:** Drug candidates for treating cryptosporidiosis in LRS.

Drug Candidate and/or Target	Developers	Funders	Current Stage	References
Clofazimine	Calibr, University of Vermont, University of Washington, Malawi-Liverpool Welcome Trust Clinical Research Programme	Bill & Melinda Gates Foundation	Terminated at Phase II clinical due to insufficient accrual rate	[11, 13, 14]
Phosphatidylinositol-4-OH kinase	Novartis, University of Georgia, Washington State University, Cornell University	Novartis, National Institutes of Health, Wellcome Trust	Preclinical	[15]
AN7973/Cleavage and Polyadenylation Specificity Factor 3	Anacor, University of Vermont, University of California San Francisco, Calibr	Bill & Melinda Gates Foundation, National Institutes of Health	Preclinical	[16, 17, 18]
Calcium-dependent protein kinase 1	University of Washington, University of Arizona, University of Bern, Calibr, AbbVie	National Institutes of Health	Preclinical	[19, 20]
Lysyl-tRNA synthetase	University of Dundee, University of Vermont, University of Washington, Calibr, GlaxoSmithKline	Bill & Melinda Gates Foundation, National Institutes of Health, Wellcome Trust, United Kingdom Medical Research Council	Preclinical	[21]
Phenylalanyl-tRNA synthetase	Broad Institute, University of Vermont, University of Pennsylvania, Calibr	Bill & Melinda Gates Foundation	Preclinical	[22]
MMV665917	University of Vermont, Tufts University, Saint Louis University, Washington State University, Calibr	Bill & Melinda Gates Foundation, National Institutes of Health	Preclinical	[23, 24, 25]
Methionyl-tRNA synthetase	University of Washington, University of Vermont, Takeda Pharmaceuticals, PATH	PATH, National Institutes of Health	Preclinical	[26]

The lack of financial return on drugs and vaccines targeting diseases that predominantly affect impoverished populations in low-income countries represents a tremendous barrier to the development of such products [27]. The FDA’s PRV program was launched in 2007 to address this issue by creating a financial incentive to develop drugs and vaccines against NTDs [28]. In brief, sponsors who receive approval for a new drug or vaccine targeting an eligible tropical disease and deemed an improvement over existing approved products can receive a PRV that entitles the holder to expedited FDA review of a future New Drug Application for any indication. Importantly, the vouchers are transferrable. PRVs have typically been purchased by pharmaceutical companies that wish to expedite review and approval of drug candidates with high commercial value, such as potential “blockbuster drugs” with projected annual sales of greater than US$1 billion. The initial list of diseases eligible for a PRV was primarily based on the World Health Organization’s list of NTDs, but there are two routes for adding new diseases to the eligibility list. The first is by an act of Congress. This has been used in two instances: the addition of Ebola and other filoviruses in 2014 and of Zika in 2016. The second is by action of the FDA, which has happened in two instances: In 2015, Chagas disease and neurocysticercosis were added, and in 2018, Chikungunya, Cryptococcal meningitis, Lassa fever, and rabies were added. FDA additions are typically in response to public comments, and, recently, a petition was submitted proposing the addition of cryptosporidiosis [29]. According to FDA guidance [30], there are two criteria for addition of a new disease to the tropical disease list: It must have “no significant market in developed nations,” and it “disproportionately affects poor and marginalized populations.” Our opinion is that cryptosporidiosis clearly meets these criteria for the reasons described below and should therefore be added to the PRV-eligible tropical disease list.

## No significant market in developed nations

Nitazoxanide was launched 17 years ago, is widely viewed as inadequate, and yet remains the only FDA-approved drug for treatment of cryptosporidiosis. There are no active clinical trials registered in the United States of investigational products to treat cryptosporidiosis (ClinicalTrials.gov search in March 2020). Similarly, no other drugs are approved for treatment of cryptosporidiosis by the European Medicines Agency, the Japanese Pharmaceutical and Medical Devices Agency, or the Australian Therapeutic Goods Administration. A major reason for the dearth of approved treatments for cryptosporidiosis in developed countries is that in these settings, the clinical course of the disease in immunocompetent and otherwise healthy individuals is usually relatively mild and self-limiting. This is in contrast to the more severe, often life-threatening clinical presentation of cryptosporidiosis among malnourished children in LRS. These facts support the conclusion of a lack of incentive for pharmaceutical companies to develop new medicines for this indication: There is an insignificant market in “developed nations.”

## Disproportionately affects poor and marginalized populations

While a recent report from the US Centers for Disease Control and Prevention (CDC) highlighted the increasing incidence of cryptosporidiosis in the United States [31], the burden among impoverished populations living in LRS remains several orders of magnitude larger. For example, within the nine-year period reported in the CDC study, there were 7,465 cases and a single fatality in the United States. This estimate represents only reported cases, and, after accounting for under-reporting and under-diagnosis, other studies have estimated the actual number of cases of cryptosporidiosis in the United States as high as 750,000 per year and the number of deaths at 4 per year [32]. In contrast, a recent analysis of GEMS data estimated approximately 7.6 million cases of cryptosporidiosis and 59,000 fatalities annually among children under 2 years old in sub-Saharan Africa and South Asia [33], substantially higher than the estimated burden among all age groups in the United States. This distinction is highlighted further by the GBD study in children under 5 years, which estimated that more than 99% of cryptosporidiosis cases and fatalities occurred in low- and middle-income countries [7]. This same study also revealed that, beyond the acute effects of cryptosporidiosis diarrhea that account for 4.2 million disability-adjusted life years (DALYs) in children under 5 years, cryptosporidiosis is responsible for an additional burden of malnutrition and growth stunting that causes another 7.85 million DALYs. This impact is almost exclusively felt in low- and middle-income countries. These findings clearly demonstrate that cryptosporidiosis meets the requirement of disproportionately affecting poor and marginalized populations.

Since release of the GEMS results in 2013, there has been renewed interest in development of more effective anticryptosporidial drugs. As a result of the efforts of various consortia, comprised of academic, nonprofit, and/or industry partners, several promising drug leads have emerged (Table 1). The quality of these leads is evidenced by active engagement of pharmaceutical companies with the capacity to advance a development program to registration, commercial-scale manufacturing, and introduction. However, pharmaceutical company investment remains limited due to the low expected return and the substantial investment still required for preclinical and clinical testing, including field testing in LRS with a high burden of disease. An incentive program has the potential to play a critical role in stimulating the advancement of one or more of these candidates. The PRV program has already been successful in attracting investment in the development of drug and vaccine candidates targeting other NTDs. For example, the Australia-based nonprofit organization Medicines Development for Global Health (MDGH) received a $13 million investment from the Global Health Investment Fund to develop a veterinary anthelmintic, moxidectin, for use in treating onchocerciasis in humans [34]. Moxidectin was approved by the FDA in 2018, and a PRV was awarded, which MDGH subsequently sold to Novo Nordisk for an undisclosed sum. Similarly, PATH secured an investment of $25 million from Clarus and the Global Health Investment Fund to lead a consortium of government, nonprofit, and commercial partners toward the development and FDA approval of tribendimidine for treatment of soil-transmitted helminths [35]. In 2015, PaxVax received an investment of $105 million from Cerebus Capital Management [36], an undisclosed portion of which was used to conduct a Phase III clinical trial of the cholera vaccine candidate CVD 103-HgR. Upon FDA approval of CVD 103-HgR (now marketed as Vaxchora™), PaxVax received a PRV, which was subsequently sold to an undisclosed buyer. These examples demonstrate the potential of PRVs to attract investment in drug and vaccine candidates that are otherwise not commercially viable. Including cryptosporidiosis on the list of PRV-eligible diseases would increase the likelihood of attracting further investment in one or more of the promising lead compounds identified in the wake of recent new epidemiological findings.

Addition of cryptosporidiosis to the PRV-eligible list of tropical diseases has the potential to accelerate the development of new therapeutics and vaccines against this deadly disease. We urge the FDA to act quickly to approve the recently submitted petition to designate cryptosporidiosis as a tropical disease and thereby incentivize advancement of the promising drug candidates that are beginning to fill the anticryptosporidial pipeline.

Readers can submit a public comment to the FDA on this topic at: https://www.regulations.gov/comment?D=FDA-2008-N-0567-0011.
